# Sequence similarity between SARS-CoV-2 nucleocapsid and multiple sclerosis-associated proteins provides insight into viral neuropathogenesis following infection

**DOI:** 10.1038/s41598-022-27348-8

**Published:** 2023-01-08

**Authors:** Camille M. Lake, Joseph J. Breen

**Affiliations:** 1 Office of Data Science and Emerging Technologies, National Institute of Allergy and Infectious Diseases, Rockville, MD USA; 2 Division of Allergy, Immunology and Transplantation, National Institute of Allergy and Infectious Diseases, Rockville, MD USA

**Keywords:** Computational models, Computational neuroscience, MHC

## Abstract

The novel coronavirus SARS-CoV-2 continues to cause death and disease throughout the world, underscoring the necessity of understanding the virus and host immune response. From the start of the pandemic, a prominent pattern of central nervous system (CNS) pathologies, including demyelination, has emerged, suggesting an underlying mechanism of viral mimicry to CNS proteins. We hypothesized that immunodominant epitopes of SARS-CoV-2 share homology with proteins associated with multiple sclerosis (MS). Using PEPMatch, a newly developed bioinformatics package which predicts peptide similarity within specific amino acid mismatching parameters consistent with published MHC binding capacity, we discovered that nucleocapsid protein shares significant overlap with 22 MS-associated proteins, including myelin proteolipid protein (PLP). Further computational evaluation demonstrated that this overlap may have critical implications for T cell responses in MS patients and is likely unique to SARS-CoV-2 among the major human coronaviruses. Our findings substantiate the hypothesis of viral molecular mimicry in the pathogenesis of MS and warrant further experimental exploration.

## Introduction

Severe Acute Respiratory Syndrome-Coronavirus 2 (SARS-CoV-2) is a novel pathogen that emerged in late 2019 as the causative agent of COVID-19. Due to the resulting global pandemic—there have been approximately 635 million documented cases worldwide^1^—as well as the novelty of the virus itself, much attention has been focused on post-viral sequalae reported in recovered patients. A significant proportion of these sequelae are related to aberrations in the central nervous system (CNS)^2^. In fact, 1 in 4 individuals with long-COVID recently reported persistent cognitive deficits and there is an emerging consensus on the significance of long-COVID as a public health burden^3^. Neurological manifestations have also been reported during acute SARS-CoV-2 infection, including encephalitis, encephalomyelitis, neuromyelitis optica spectrum disorder, myelin oligodendrocyte glycoprotein antibody-associated disease, and multiple sclerosis^4^. Taken together, these manifestations suggest a pathogenesis potentially involving demyelination, which may further suggest the centrality of an autoimmune process in both acute and post-infectious clinical presentations in some COVID-19 patients.

Multiple sclerosis (MS) is the most common autoimmune demyelinating disease in the United States and affects approximately 3 million people worldwide^5^. It has been widely suggested that the etiology of MS involves an initial infectious insult. For decades, it has been noted that the onset of first and recurring episodes of MS are often preceded by acute infections^6–8^. Importantly, studies investigating seasonal coronaviruses and SARS-CoV-2 have suggested the ability of these viruses to elicit cross-reactivity with viruses thought to play a role in the initial pathogenesis of MS^9^. In addition, other notable autoimmune diseases such as Guillain–Barre Syndrome, hypothesized to be driven in part by molecular mimicry, have been reported following SARS-CoV-2 infection^10^. We hypothesized that SARS-CoV-2 proteins share homology with CNS proteins, which could play a role in the physical manifestations of MS following acute SARS-CoV-2 infection.

Although the development of MS is likely a coalescence of the cellular and humoral adaptive immune arms, T cells have been implicated as being central drivers of disease manifestation in both the mouse model of MS (experimental autoimmune encephalitis, EAE) and humans^11,12^. Central to the theory of molecular mimicry is the degenerative nature of both T cells and MHC binding, in which multiple peptides can bind to the same MHC, and in turn multiple peptide:MHC combinations can be recognized by the same T cell Receptor (TCR)^13,14^. Several pathogenic proteins have been assessed for their homology to MS-associated antigens^15,16^, but no analyses to date have fully captured physiological parameters like MHC degeneracy and peptide length restrictions to provide a comprehensive picture of homology in the context of MHC presentation.

Here, we use PEPMatch^17^, a newly developed, bioinformatic homology-based package, to assess the potential for molecular mimicry between known immunodominant proteins from SARS-CoV-2 and MS-associated proteins in the context of a T-cell mediated response. Critically, PEPMatch has built-in parameters for both peptide length and mismatching rate, which more closely mimics the constraints imposed by MHC presentation of peptides to TCRs and thus the potential for molecular mimicry. We report that nucleocapsid protein from SARS-CoV-2 shares significant overlap with MS-associated proteins, including the canonical MS protein, myelin proteolipid protein (PLP). Our computational study substantiates the molecular mimicry hypothesis in the neurological sequelae of SARS-CoV-2 infection and lays the groundwork for future experimental and epidemiological studies investigating MS pathogenic etiologies.

## Results

### SARS-CoV-2 nucleocapsid exhibits significant homology with MS-associated proteins across both 9mer and 15mer peptide groups

**Figure 1:**
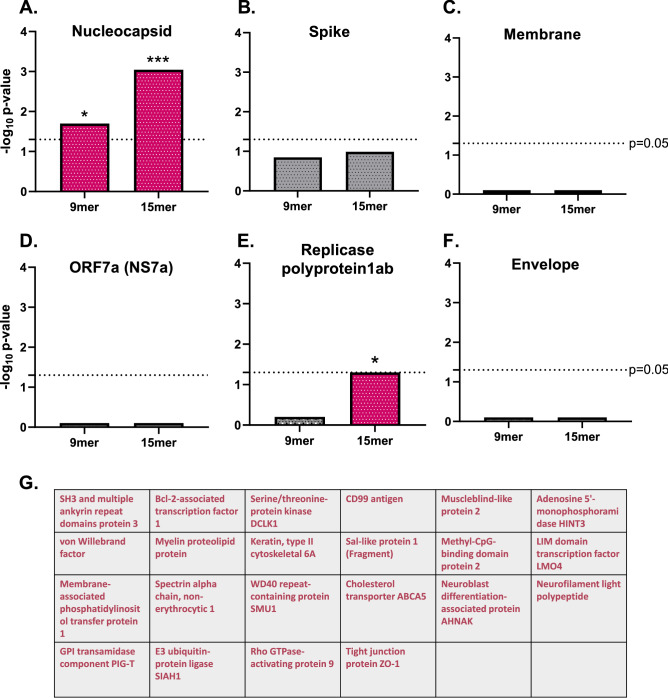
Nucleocapsid protein shares significant homology with MS-associated proteins. (**A**) PEPMatch was used to determine the overlap between nucleocapsid protein from SARS-CoV-2 and MS-associated proteins, which were determined using an IEDB query. For comparison, 30 iterations of shuffled-sequence nucleocapsid protein were run through the analysis with the same parameters as the intact nucleocapsid sequence, whose average was then compared to MS-associated proteins. Fisher’s exact or Chi square tests were run on both the 9mer and the 15mer peptides, with up to 2 mismatches for the 9mer peptides and up to 7 mismatches for the 15mer peptides. (**B**–**F**) The same analysis was run as in (**A**) using the specified proteins labeled above each graph from SARS-CoV-2. (**G**) Shown are the proteins whose peptides significantly overlapped with nucleocapsid across both the 9mer and 15mer groups.

We used PEPMatch^17^ to determine the sequence homology between immunodominant proteins from SARS-CoV-2 and MS-associated proteins, which were compiled using the Immune Epitope Database and Analysis Resource (IEDB) (http://www.iedb.org/) (Table 1). We tested both 9mer and 15mer segments to contextualize MHC I and MHC II presentation, respectively. These peptide lengths correspond to experimentally validated epitope lengths associated with CD8^+^ and CD4^+^ T cell recognition, respectively^17^. Intriguingly, nucleocapsid protein shared significant overlap with MS-associated proteins in both 9mer and 15mer groups (Fig. 1A) (see Methods for full details on background controls and statistical comparisons). Spike, membrane, NS7a, and envelope proteins from SARS-CoV-2 did not share significant overlap with the list of MS proteins above their sequence-shuffled controls, and replicase polyprotein 1ab had significantly elevated peptide matches above its sequence-shuffled control only in the 15mer group (Fig. 1B–F). Out of the 108 antigens on the list of MS-associated proteins used in this analysis, 22 proteins had sequences matching nucleocapsid across both 9mer and 15mer groups. Of note, the canonical MS-associated protein PLP shared homology with nucleocapsid from SARS-CoV-2 (Fig. 1G).

**Table 1:** List of proteins used in this analysis.

UniProt IDs	Protein name	Gene names	Organism
Q07157	Tight junction protein ZO-1	TJP1	Homo sapiens (Human)
Q9Y6M0	Testisin	PRSS21	Homo sapiens (Human)
P63313	Thymosin beta-10	TMSB10	Homo sapiens (Human)
Q9Y617	Phosphoserine aminotransferase	PSAT1	Homo sapiens (Human)
Q01082	Spectrin beta chain, non-erythrocytic 1	SPTBN1 SPTB2	Homo sapiens (Human)
Q6ZMD2	Protein spinster homolog 3	SPNS3	Homo sapiens (Human)
Q8IUQ4	E3 ubiquitin-protein ligase SIAH1	SIAH1 HUMSIAH	Homo sapiens (Human)
O94885	SAM and SH3 domain-containing protein 1	SASH1 KIAA0790 PEPE1	Homo sapiens (Human)
P04275	von Willebrand factor	VWF F8VWF	Homo sapiens (Human)
Q16864	V-type proton ATPase subunit F	ATP6V1F ATP6S14 VATF	Homo sapiens (Human)
O43660	Pleiotropic regulator 1	PLRG1	Homo sapiens (Human)
P20132	L-serine dehydratase/L-threonine deaminase	SDS SDH	Homo sapiens (Human)
Q06190	Serine/threonine-protein phosphatase 2A regulatory subunit B'' subunit alpha	PPP2R3A PPP2R3	Homo sapiens (Human)
Q70J99	Protein unc-13 homolog D	UNC13D	Homo sapiens (Human)
Q9NYF8	Bcl-2-associated transcription factor 1	BCLAF1 BTF KIAA0164	Homo sapiens (Human)
P05023	Sodium/potassium-transporting ATPase subunit alpha-1	ATP1A1	Homo sapiens (Human)
Q6JQN1	Acyl-CoA dehydrogenase family member 10	ACAD10	Homo sapiens (Human)
Q8WWZ7	Cholesterol transporter ABCA5	ABCA5 KIAA1888	Homo sapiens (Human)
Q8N6D5	Ankyrin repeat domain-containing protein 29	ANKRD29	Homo sapiens (Human)
Q9BZR8	Apoptosis facilitator Bcl-2-like protein 14	BCL2L14 BCLG	Homo sapiens (Human)
Q9H0Q3	FXYD domain-containing ion transport regulator 6	FXYD6 UNQ521/PRO1056	Homo sapiens (Human)
Q9HC77	Centromere protein J	CENPJ CPAP LAP LIP1	Homo sapiens (Human)
P25024	C-X-C chemokine receptor type 1	CXCR1 CMKAR1 IL8RA	Homo sapiens (Human)
P63151	Serine/threonine-protein phosphatase 2A 55 kDa regulatory subunit B alpha isoform	PPP2R2A	Homo sapiens (Human)
Q14469	Transcription factor HES-1	HES1 BHLHB39 HL HRY	Homo sapiens (Human)
Q09666	Neuroblast differentiation-associated protein AHNAK	AHNAK PM227	Homo sapiens (Human)
Q12860	Contactin-1	CNTN1	Homo sapiens (Human)
P49771	Fms-related tyrosine kinase 3 ligand	FLT3LG	Homo sapiens (Human)
Q9Y6R7	IgGFc-binding protein	FCGBP	Homo sapiens (Human)
P14209	CD99 antigen	CD99 MIC2 MIC2X MIC2Y	Homo sapiens (Human)
P04083	Annexin A1	ANXA1 ANX1 LPC1	Homo sapiens (Human)
P0DP25	Calmodulin-3	CALM3 CALML2 CAM3 CAMC CAMIII	Homo sapiens (Human)
P50995	Annexin A11	ANXA11 ANX11	Homo sapiens (Human)
Q7L5A8	Fatty acid 2-hydroxylase	FA2H FAAH FAXDC1	Homo sapiens (Human)
Q9UBB5	Methyl-CpG-binding domain protein 2	MBD2	Homo sapiens (Human)
Q96KP4	Cytosolic non-specific dipeptidase	CNDP2 CN2 CPGL HEL-S-13 PEPA	Homo sapiens (Human)
O15075	Serine/threonine-protein kinase DCLK1	DCLK1 DCAMKL1 DCDC3A KIAA0369	Homo sapiens (Human)
Q9NPB8	Glycerophosphocholine phosphodiesterase GPCPD1	GPCPD1 GDE5 KIAA1434	Homo sapiens (Human)
P50502	Hsc70-interacting protein	ST13 AAG2 FAM10A1 HIP SNC6	Homo sapiens (Human)
P07099	Epoxide hydrolase 1	EPHX1 EPHX EPOX	Homo sapiens (Human)
P61968	LIM domain transcription factor LMO4	LMO4	Homo sapiens (Human)
Q5QNW6	Histone H2B type 2-F	H2BC18 HIST2H2BF	Homo sapiens (Human)
P01344	Insulin-like growth factor II	IGF2 PP1446	Homo sapiens (Human)
A4QPB2	Low-density lipoprotein receptor-related protein 5-like protein	LRP5L	Homo sapiens (Human)
P05783	Keratin, type I cytoskeletal 18	KRT18 CYK18 PIG46	Homo sapiens (Human)
Q9UBV8	Peflin	PEF1 ABP32 UNQ1845/PRO3573	Homo sapiens (Human)
Q92903	Phosphatidate cytidylyltransferase 1	CDS1 CDS	Homo sapiens (Human)
P49327	Fatty acid synthase	FASN FAS	Homo sapiens (Human)
P20592	Interferon-induced GTP-binding protein Mx2	MX2	Homo sapiens (Human)
P60201	Myelin proteolipid protein	PLP1 PLP	Homo sapiens (Human)
P60709	Actin, cytoplasmic 1	ACTB	Homo sapiens (Human)
Q14938	Nuclear factor 1 X-type	NFIX	Homo sapiens (Human)
Q9H1E3	Nuclear ubiquitous casein and cyclin-dependent kinase substrate 1	NUCKS1 NUCKS JC7	Homo sapiens (Human)
P20292	Arachidonate 5-lipoxygenase-activating protein	ALOX5AP FLAP	Homo sapiens (Human)
Q6ZMW3	Echinoderm microtubule-associated protein-like 6	EML6 EML5L	Homo sapiens (Human)
P09471	Guanine nucleotide-binding protein G(o) subunit alpha	GNAO1	Homo sapiens (Human)
Q13349	Integrin alpha-D	ITGAD	Homo sapiens (Human)
Q13491	Neuronal membrane glycoprotein M6-b (M6b)	GPM6B M6B	Homo sapiens (Human)
Q5VZF2	Muscleblind-like protein 2	MBNL2 MBLL MBLL39 MLP1	Homo sapiens (Human)
O95897	Noelin-2	OLFM2 NOE2	Homo sapiens (Human)
O00562	Membrane-associated phosphatidylinositol transfer protein 1	PITPNM1 DRES9 NIR2 PITPNM	Homo sapiens (Human)
Q9NQE9	Adenosine 5'-monophosphoramidase HINT3	HINT3	Homo sapiens (Human)
P02686	Myelin basic protein (P02868)	MBP	Homo sapiens (Human)
P55082	Microfibril-associated glycoprotein 3	MFAP3	Homo sapiens (Human)
P07196	Neurofilament light polypeptide	NEFL NF68 NFL	Homo sapiens (Human)
Q8IXS6	Paralemmin-2	PALM2	Homo sapiens (Human)
Q6NY19	KN motif and ankyrin repeat domain-containing protein 3	KANK3 ANKRD47	Homo sapiens (Human)
P20916	Myelin-associated glycoprotein	MAG GMA	Homo sapiens (Human)
O95298	NADH dehydrogenase [ubiquinone] 1 subunit C2	NDUFC2 HLC1	Homo sapiens (Human)
P68871	Hemoglobin subunit beta	HBB	Homo sapiens (Human)
Q9H2D1	Mitochondrial folate transporter/carrier	SLC25A32 MFT MFTC	Homo sapiens (Human)
Q13813	Spectrin alpha chain, non-erythrocytic 1	SPTAN1 NEAS SPTA2	Homo sapiens (Human)
Q6UWS5	Protein PET117 homolog, mitochondrial	PET117 UNQ607/PRO1194	Homo sapiens (Human)
Q8IXJ6	NAD-dependent protein deacetylase sirtuin-2	SIRT2 SIR2L SIR2L2	Homo sapiens (Human)
P0DTU4	T cell receptor beta chain MC.7.G5	TRB	Homo sapiens (Human)
P37837	Transaldolase	TALDO1 TAL TALDO TALDOR	Homo sapiens (Human)
P04271	Protein S100-B	S100B	Homo sapiens (Human)
O00193	Small acidic protein	SMAP C11orf58	Homo sapiens (Human)
Q2TAY7	WD40 repeat-containing protein SMU1	SMU1	Homo sapiens (Human)
P62273	40S ribosomal protein S29	RPS29	Homo sapiens (Human)
P02538	Keratin, type II cytoskeletal 6A	KRT6A K6A KRT6D	Homo sapiens (Human)
P35579	Myosin-9	MYH9	Homo sapiens (Human)
P16949	Stathmin	STMN1 C1orf215 LAP18 OP18	Homo sapiens (Human)
Q7KZF4	Staphylococcal nuclease domain-containing protein 1	SND1 TDRD11	Homo sapiens (Human)
Q86YJ6	Threonine synthase-like 2	THNSL2	Homo sapiens (Human)
Q15437	Protein transport protein Sec23B	SEC23B	Homo sapiens (Human)
E7EV99	Alpha-adducin	ADD1	Homo sapiens (Human)
X6RJP6	Transgelin-2 (Fragment)	TAGLN2	Homo sapiens (Human)
A0A0A0MS51	Actin-depolymerizing factor	GSN	Homo sapiens (Human)
C9J9K3	40S ribosomal protein SA (Fragment)	RPSA	Homo sapiens (Human)
J3QQK6	Myelin basic protein (J3QQK6)	MBP	Homo sapiens (Human)
H3BQR2	Cytosolic Fe-S cluster assembly factor NUBP2	NUBP2	Homo sapiens (Human)
B5MCX3	Septin-2	SEPTIN2	Homo sapiens (Human)
B7WPG3	Heterogeneous nuclear ribonucleoprotein L-like	HNRNPLL	Homo sapiens (Human)
F5H5N1	Complex I-20kD	NDUFS7	Homo sapiens (Human)
E9PEF9	Aldo–keto reductase family 1 member B1	AKR1B1	Homo sapiens (Human)
A0A0U1RQS4	SH3 and multiple ankyrin repeat domains protein 3	SHANK3	Homo sapiens (Human)
H3BSM9	Sal-like protein 1 (Fragment)	SALL1	Homo sapiens (Human)
Q6FGG4	Complex I-B9	NDUFA3 hCG_20947	Homo sapiens (Human)
J3KPH8	Histone deacetylase	HDAC7	Homo sapiens (Human)
R4GN15	Rho GTPase-activating protein 9	ARHGAP9	Homo sapiens (Human)
A0A1W2PP57	GPI transamidase component PIG-T	PIGT	Homo sapiens (Human)
C9JVQ0	Small nuclear ribonucleoprotein G	SNRPG	Homo sapiens (Human)
H0Y4W2	Transformation/transcription domain-associated protein	TRRAP	Homo sapiens (Human)
X1WI28	60S ribosomal protein L10 (Fragment)	RPL10	Homo sapiens (Human)
E9PB61	THO complex subunit 4	ALYREF	Homo sapiens (Human)
J3KN36	Nodal modulator 3	NOMO3	Homo sapiens (Human)
Q16653	Myelin oligodendrocyte-glycoprotein	MOG	Homo sapiens (Human)
P0DTC2	Spike glycoprotein	S	Severe acute respiratory syndrome coronavirus 2
A0A6C0T6Z7	Nucleoprotein (Nucleocapsid)	N	Severe acute respiratory syndrome coronavirus 2
P0DTC5	Membrane protein	M	Severe acute respiratory syndrome coronavirus 2
P15423	Spike glycoprotein	S	Human coronavirus 229E
P15130	Nucleoprotein (Nucleocapsid)	N	Human coronavirus 229E
P15422	Membrane protein	M	Human coronavirus 229E
Q6Q1S2	Spike glycoprotein	S	Human coronavirus NL63
Q6Q1R8	Nucleoprotein (Nucleocapsid)	N	Human coronavirus NL63
Q6Q1R9	Membrane protein	M	Human coronavirus NL63
P36334	Spike glycoprotein	S	Human coronavirus OC43
P33469	Nucleoprotein (Nucleocapsid)	N	Human coronavirus OC43
Q01455	Membrane protein	M	Human coronavirus OC43
Q5MQD0	Spike glycoprotein	S	Human coronavirus HKU1 (isolate N1)
Q5MQC6	Nucleoprotein (Nucleocapsid)	N	Human coronavirus HKU1 (isolate N1)
Q5MQC7	Membrane protein	M	Human coronavirus HKU1 (isolate N1)
P03211	Epstein-Barr nuclear antigen 1	EBNA1	Epstein-Barr virus (strain B95-8)
P12978	Epstein-Barr nuclear antigen 2	EBNA2	Epstein-Barr virus (strain B95-8)
P12977	Epstein-Barr nuclear antigen 3	EBNA3	Epstein-Barr virus (strain B95-8)
P03203	Epstein-Barr nuclear antigen 4	EBNA4	Epstein-Barr virus (strain B95-8)
Q8AZK7	Epstein-Barr nuclear antigen leader protein	EBNA-LP	Epstein-Barr virus (strain B95-8)
P03204	Epstein-Barr nuclear antigen 6	EBNA6	Epstein-Barr virus (strain B95-8)
P03230	Latent membrane protein 1	LMP1	Epstein-Barr virus (strain B95-8)
P13285	Latent membrane protein 2	LMP2	Epstein-Barr virus (strain B95-8)
P06725	65 kDa phosphoprotein	UL83	Human cytomegalovirus (strain AD169)
P13202	Immediate early protein IE1	UL123	Human cytomegalovirus (strain AD169)
P06473	Envelope glycoprotein B	gB	Human cytomegalovirus (strain AD169)
P09713	Unique short US2 glycoprotein	US2	Human cytomegalovirus (strain AD169)
P13200	Cytoplasmic envelopment protein 3	UL99	Human cytomegalovirus (strain AD169)
P12824	Envelope glycoprotein H	gH	Human cytomegalovirus (strain AD169)
P09712	Membrane glycoprotein US3	US3	Human cytomegalovirus (strain AD169)
P14334	Unique short US6 glycoprotein	US6	Human cytomegalovirus (strain AD169)
P08560	Glycoprotein UL18	H301	Human cytomegalovirus (strain AD169)
P0DTC7	ORF7a protein	7a	Severe acute respiratory syndrome coronavirus 2
P0DTD1	Replicase polyprotein 1ab	Rep	Severe acute respiratory syndrome coronavirus 2
P0DTC4	Envelope small membrane protein	E	Severe acute respiratory syndrome coronavirus 2

Briefly, MS-associated proteins were compiled using the IEDB (see Methods for full methodology on protein selection). This was to ensure that proteins utilized in this analysis have been experimentally associated with MS in the literature. SARS-CoV-2, seasonal coronavirus, EBV, and CMV immunodominant proteins are also listed.

### PEPMatch-predicted PLP epitope shares homology with experimentally validated MS-associated peptides and is in a region known to elicit T-cell responses in MS patients

**Figure 2:**
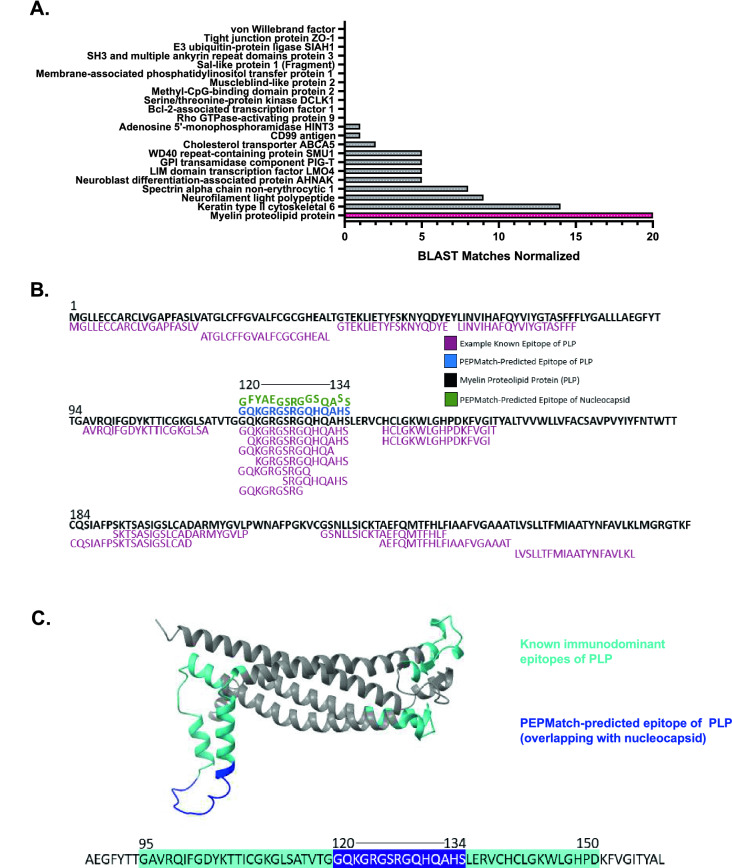
PEPMatch-predicted epitope of myelin proteolipid protein (PLP) has high similarity to experimentally validated epitopes and is in a region associated with strong T cell responses in MS patients. (**A**) PEPMatch-predicted peptides sharing homology with nucleocapsid were assessed for overlap with known, experimentally validated epitopes on the Immune Epitope Databases (IEDB). All known epitopes for each protein were run in a BLASTp query against the peptide(s) returned from PEPMatch. The number of homology “hits” were quantified and divided by the number of input PEPMatch peptides for that protein for normalization. (**B**) The PEPMatch-predicted epitope of PLP matching nucleocapsid (blue) was aligned with the full-length sequence of PLP (black) and the nucleocapsid peptide to which it matches (green) alongside examples of known epitopes of PLP identified in the literature and available on the IEDB (berry). (**C**) Predicted PLP structure was highlighted with known immunodominant areas previously identified as eliciting strong T cell responses preferentially in MS patients^20^ (cyan) against the PEPMatch-predicted epitope sharing homology with nucleocapsid (dark blue).

To contextualize the above findings, we investigated whether epitopes returned from PEPMatch had been documented in the literature. We found that of all the proteins returned from the PEPMatch analysis, PLP had the highest number of BLASTp-verified homologous sequences that have been experimentally validated and curated on the IEDB (Fig. 2A,B). PLP has been documented to contain epitopes recognized by T cells from MS patients across numerous studies^18,19^. In one study, T cell lines derived from MS patients and activated with PLP showed the strongest reactivity against regions 40–60, 95–117, 117–150, and 185–206 out of all 9 regions of PLP tested^20^. The authors concluded from this study that these regions were largely responsible for eliciting strong T cell responses in MS patients. We found that the PLP peptides returned from PEPMatch fell within one of these immunodominant regions (Fig. 2C). Overall, these results provide a computational basis for the potential of SARS-CoV-2 to initiate T-cell-driven molecular mimicry through specific MS-associated proteins, including PLP.

### MHC-binding prediction reveals other proteins of interest which may facilitate molecular mimicry beyond PLP

**Figure 3:**
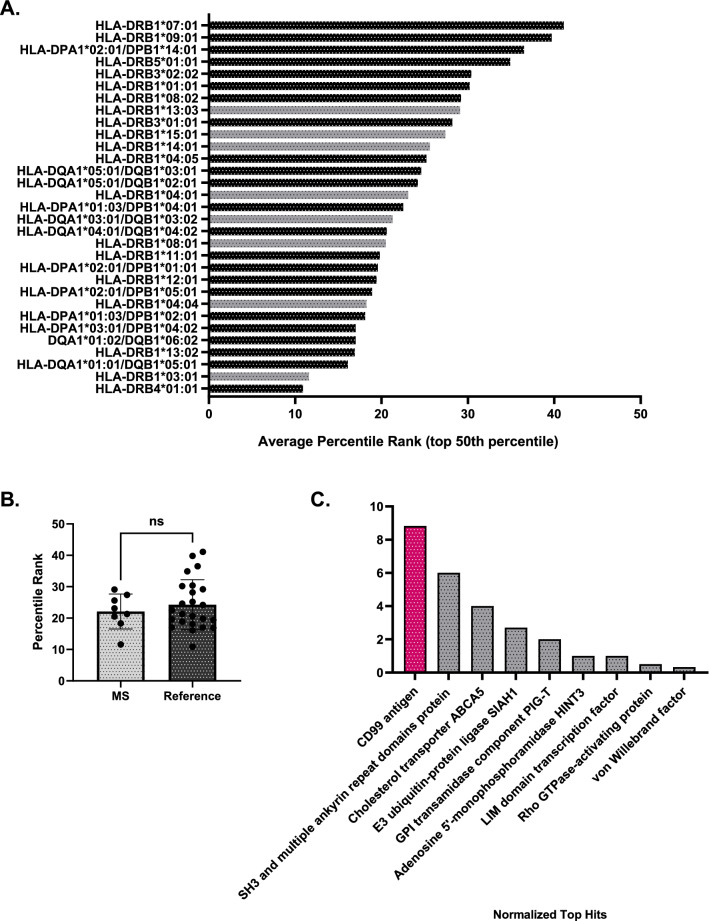
MHC-binding prediction provides insight on proteins which may engage in molecular mimicry beyond PLP. (**A**) MHC-binding prediction tool by the IEDB was used to determine the binding of nucleocapsid peptide hits from the PEPMatch analysis and MS-associated alleles (gray) versus a reference set of alleles (black)^25^. Shown are the top 50th percentile of average binding predictions of all 15mer nucleocapsid peptides from the original analysis and their respective HLA alleles that are predicted to bind. The lower the percentile rank, the stronger the predicted binding between the allele and the set of peptides run in the analysis. (**B**) Data from (**A**) grouped by allele classification. (**C**) Peptide:allele combinations with a percentile rank of 10th or less were collected and assessed for their respective MS-associated protein matches from the original PEPMatch analysis; plotted are the proteins that were returned from this analysis that are normalized to their respective number of peptides input.

Given that molecular mimicry is likely facilitated not only by sequence similarity but also by HLA haplotype^6,21^, we next investigated whether MS-associated alleles^22–24^ would be predicted to bind to the nucleocapsid peptides returned from the PEPMatch analysis. We compared the binding propensity of MS-associated alleles to a set of alleles which represent approximately 99% of the population worldwide to contextualize the analysis^25^. We analyzed the top 50th percentile of binding predictions returned from the algorithm in order to focus on physiologically relevant binding predictions while retaining maximum information on the alleles^26^. We found that while some MS-associated alleles demonstrated high average binding capacities (represented by low average percentile rank, Fig. 3A), including HLA-DRB1*03:01, HLA-DRB1*04:04, and HLA-DRB1*08:01, there was no significant difference in average binding predictions from MS-associated alleles in comparison to the reference set of alleles (Fig. 3B). We next asked whether certain proteins matching the nucleocapsid peptides from the original PEPMatch analysis were enriched amongst the top peptide:allele binding predictions. Inspection of these peptide:allele combinations that scored a 10th percentile rank or less across all alleles revealed unique proteins enriched for predicted MHC binding (Fig. 3C). Although our focus has largely centered on the canonical MS protein PLP, this analysis highlights other potential proteins returned from the PEPMatch analysis that may trigger autoimmunity across a wide variety of HLA-haplotyped individuals, including CD99 (Fig. 3C).

### Seasonal coronaviruses share significant homology with MS-associated proteins but do not overlap with PLP

**Figure 4:**
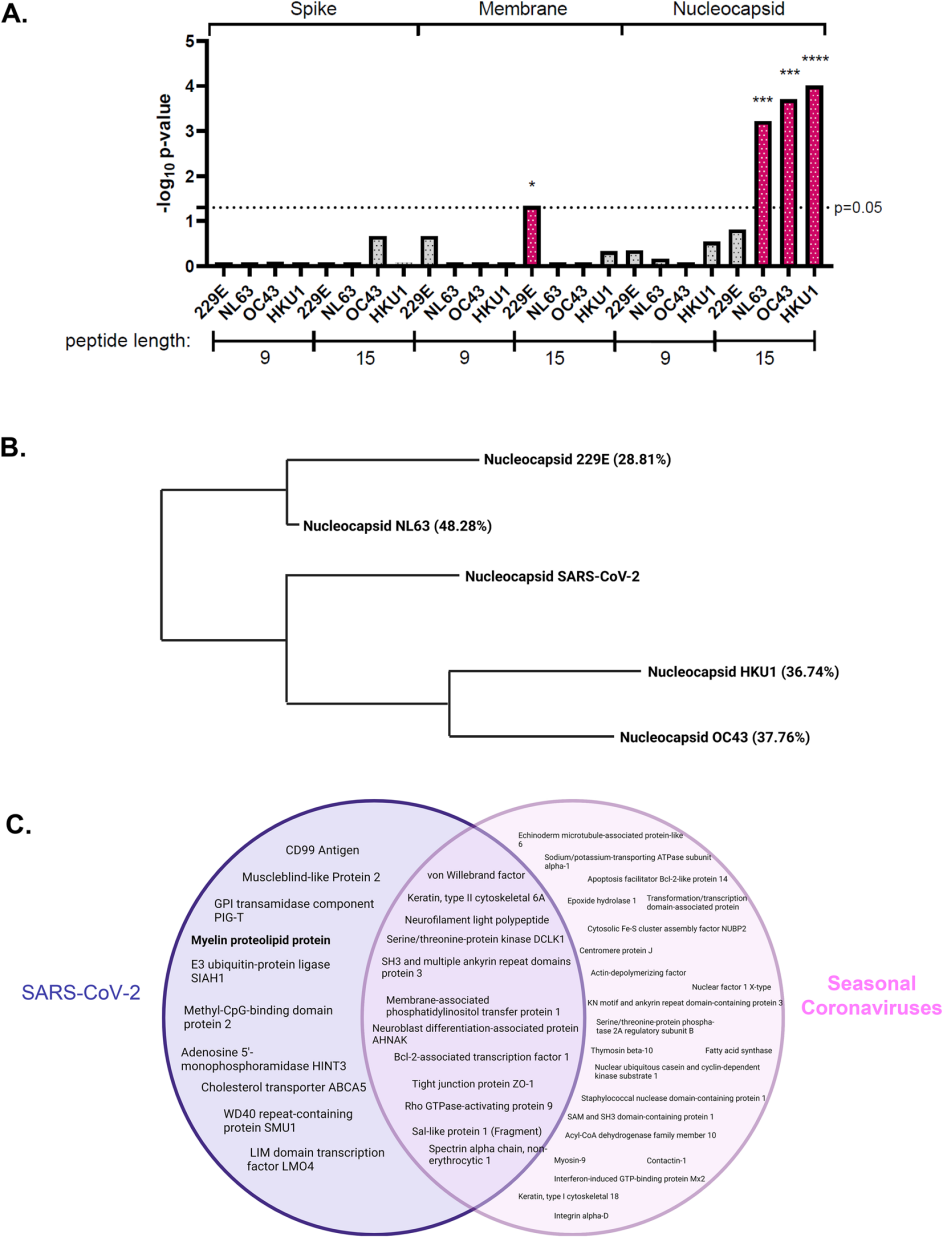
Nucleocapsid of seasonal coronaviruses show homology with MS-associated proteins, but only SARS-CoV-2 overlaps with PLP. (**A**) PEPMatch homology analysis was conducted as in Fig. 1 using spike, membrane, and nucleocapsid proteins from seasonal coronaviruses 229E, NL63, OC43, and HKU1. (**B**) BLASTp analysis was conducted to determine overall percent identity of seasonal coronavirus nucleocapsid proteins against SARS-CoV-2 nucleocapsid. Shown is a representation of the BLASTp homology output, with percent identity to SARS-CoV-2 nucleocapsid in parentheses. (**C**) PEPMatch-predicted proteins sharing homology with SARS-CoV-2 (left) and seasonal coronaviruses (right) were assessed for overlap using a Venn diagram.

Seasonal coronaviruses have been implicated in the development of MS^27–29^. To determine whether the above findings were unique to SARS-CoV-2, we tested whether nucleocapsid, spike, or membrane proteins from seasonal coronaviruses 229E, NL63, OC43, and HKU1 shared significant homology with MS-associated proteins. We found that the nucleocapsid protein from 3 out of the 4 seasonal coronaviruses tested shared significant homology with MS-associated proteins, but only in the 15mer groups (Fig. 4A); due to the stricter matching parameters of the 9mer group, this suggests a higher degree of peptide similarity between SARS-CoV-2 nucleocapsid and MS-associated proteins (Fig. 1A). Nucleocapsid proteins of these 3 seasonal coronaviruses also share the highest percent identity to SARS-CoV-2 nucleocapsid as determined by BLASTp (Fig. 4B). Of note, while the coronaviruses all had some shared PEPMatch protein “hits”, only PLP significantly overlapped with the nucleocapsid of SARS-CoV-2 (Fig. 4C).

## Discussion

Since the beginning of the pandemic, SARS-CoV-2 has been associated with CNS sequelae with manifestations ranging from memory loss and attention deficits to demyelination^3,4,30,31^. Although viral molecular mimicry has been a long-standing hypothesis regarding the triggering of initial and recurrent episodes of MS demyelination^7^, no bioinformatic approaches had been created which consider physiological parameters necessary for fully understanding MHC presentation capacity. In this analysis, we demonstrated that SARS-CoV-2 may be associated with the development of MS using a new computational tool developed by the IEDB team that includes more robust physiological parameters in its assessment for homology.

SARS-CoV-2 nucleocapsid, spike, and membrane proteins have been widely demonstrated to elicit strong immune responses^32^. Indeed, it was recently found that these 3 proteins were among the 9 viral proteins making up 83% of CD4^+^ T cell responses, and among the 8 accounting for 81% of CD8^+^ T cell responses in COVID-convalescent patients^33,34^. In addition, NS7a^35^, replicase polyprotein1ab^36^, and envelope^37^ proteins have all been implicated in driving immune responses in individuals recovering from SARS-CoV-2. We asked whether any of these proteins shared significant homology with MS-associated neuro-antigens—an observation which would fortify the proposed case for molecular mimicry in the development of MS following SARS-CoV-2 infection. Interestingly, both the 9mer and 15mer groups (representing MHC I and II, respectively) from nucleocapsid showed significant sequence overlap with MS-associated proteins (Fig. 1A), in contrast to most of the other proteins tested in our analysis with the exception of replicase polyprotein 1ab, which showed significance only in the 15mer group (Fig. 1E). Given the strict parameters for amino acid homology for the 9mer group—protein sequences had to exactly match a minimum of 78% of the time—a strong overlap of nucleocapsid and MS-associated proteins highlights the potential for SARS-CoV-2-mediated molecular mimicry across both classes of MHC.

Myelin proteolipid protein (PLP) has been implicated in the development of MS across a multitude of studies^20,38–40^. In humans, the development of MS following Rubella virus infection was demonstrated to be linked to the high relative similarity score of E2 protein to PLP^16,41^, demonstrating the potential for sequence overlap leading to the induction of demyelinating disease. More recently, França et al. discovered that the NS5 epitope of Zika virus shared 83% sequence homology with PLP, implicating NS5 as a likely candidate for driving the development of MS and possibly other CNS inflammatory demyelinating disorders^42^. In our own study using PEPMatch, we found that an epitope from PLP shared significant homology with a nucleocapsid peptide from SARS-CoV-2 (Fig. 1G). Importantly, the PLP epitope that overlaps with nucleocapsid is associated with a high number of experimentally validated epitopes curated from the literature (Fig. 2A,B), providing evidence for the potential for SARS-CoV-2-driven molecular mimicry. Among the numerous studies that have investigated the autoantigenicity of specific epitopes of PLP in the context of MS-development^18,19^, certain epitopes have been specifically associated with eliciting strong T cell responses in MS patients^20^. The epitope returned from PEPMatch in our study, 120–134, is encompassed within an epitope associated with strong T cell responses in DR15*01-positive MS subjects^20^ (Fig. 2C). Collectively, this data substantiates the hypothesis that SARS-CoV-2 nucleocapsid may be providing the basis for molecular mimicry preceding the development of MS in susceptible individuals.

In the words of Wekerle, “Molecular mimicry thus goes well beyond the simple structural resemblance of two individual peptides. It also embraces the peptide-presenting MHC…”^7^. We investigated whether nucleocapsid peptides returned from PEPMatch would be predicted to bind preferentially to MS-associated class II HLA alleles, of which more has been published in relation to MS susceptibility than for class I HLA. Using the top 50th percentile of binding predictions, the average percentile rank of all peptide:allele combinations grouped by allele demonstrated no significant pattern of binding enrichment among the MS-associated alleles (Fig. 3B). Several salient considerations must be acknowledged when interpretating these data, however. Importantly, weakly binding peptide epitopes from myelin basic protein (MBP) have been associated with eliciting strong autoimmune responses in EAE mouse models^43^, suggesting that predicting the binding propensity of peptide:allele combinations may be more complex than fully accounted for by this machine learning algorithm. This analysis also is limited in scope by the total number of HLA alleles assessed. We analyzed 31 alleles; there are over 33,000 allele and haplotype entries on the IPD/IMGT-HLA database^44^. However, our results suggest that certain HLA alleles not previously associated with MS development may increase susceptibility to CNS-related sequelae, including HLA-DRB4*01:01, though this speculation warrants further investigation.

Top ranking MS-associated alleles from our analysis, including HLA-DRB1*03:01, have not only been associated with the development of MS, but also the development of other autoimmune disorders. HLA-DRB1*03:01 has been associated with autoimmune hepatitis^45^, autoimmune encephalitis^46^, neuromyelitis optica^47^, and autoimmune Addison’s disease (AAD)^48^. AAD has been reported in the literature following acute COVID-19 infection^49–51^, suggesting that this allele may predispose individuals of this haplotype to other acute autoimmune-related manifestations of SARS-CoV-2 beyond CNS pathologies. In conclusion, numerous studies have been published which link specific HLA haplotypes with susceptibility of severe COVID outcomes^52–54^; however, no studies exist to date which explore the haplotypes of individuals with rare CNS sequelae following SARS-CoV-2 infection. Future experimental investigation should broaden the scope of disease manifestations to better understand the potential link between HLA haplotype and SARS-CoV-2 neuropathogenesis.

To further explore the relationship between allele binding predictions and CNS manifestations, we gathered the peptides of percentile rank 10 or lower^26^ and tallied the original proteins from which the peptides originated. We found a considerable enrichment of CD99 peptides among the top allele binding predictions (Fig. 3C). CD99 is a cell surface protein expressed by a wide number of tissues and organ systems, including lymphocytes, and is critical for cellular adhesion, migration, and diapedesis^55^. Recently, CD99 was implicated in exacerbated COVID-19-associated kidney injury^56^. Elution of CD99 peptides in the urine separated groups of patients with mild versus severe kidney pathology; in addition, CD99^+^ lymphocytes were found in significantly lower percentages in patients with severe outcomes. The authors speculated that aberrant autoimmune responses directed against CD99 may be promoting the exacerbation of kidney injury, and that this reduction of CD99 overall could indicate a loss of endothelial integrity^56^. Recently, Domizio and colleagues found that acute respiratory injury following SARS-CoV-2 infection is in part due to lung endothelial damage, which they speculated translated to other organ systems as well^57^. Neuropathogenesis following SARS-CoV-2 infection has been noted for distinct pathophysiological patterns, including loss of blood–brain barrier (BBB) integrity^58^, which is structurally maintained largely through endothelial cells^59^. Integral to these observations is the finding that Keratin Type II Cytoskeleton 6A (a protein recovered in our original PEPMatch analysis, Fig. 1G) was also found to be dysregulated in patients with severe kidney injury^56^. This keratin protein has been associated with wound healing, in which loss of this protein led to profound inabilities of mice to undergo normal wound healing processes^60^. The peptides identified from PEPMatch (Fig. 1G), and more specifically those predicted to be bound and presented on MHC (Fig. 3C) warrant further experimental investigation, as they collectively suggest a mechanism by which molecular mimicry initiated by SARS-CoV-2 infection could lead to the inappropriate targeting of proteins involved in a range of biological processes necessary for a multitude of organ systems, culminating in severe neurological and other pathological outcomes.

Molecular mimicry has been explored as a mechanism for triggering MS for decades, and several viral and bacterial pathogens have been associated with MS development^61^. The most strongly linked etiological agent of MS is Epstein-Barr virus (EBV), in which multiple recent studies have strongly implicated the development of MS with EBV infection^62,63^. In our own hands using PEPMatch, we were able to demonstrate that a larger proportion of EBV proteins share similarity with MS-associated proteins than its virologic cousin, cytomegalovirus (CMV), aligning with current views on MS etiology and substantiating the practicality and utility of PEPMatch as a new resource (Supplemental Fig. 1). Seasonal coronaviruses have also been implicated in the pathogenesis of MS using a range of bioinformatic and clinical approaches^27–29^. Primary T cell clones isolated from MS patients activated with HCoV-229E and HCoV-OC43 proteins cross-reacted with myelin basic protein (MBP) and PLP, highlighting the propensity for viral molecular mimicry involving seasonal coronaviruses^29^. In our study, we found that nucleocapsid protein from 3 out of the 4 major seasonal coronaviruses showed significant sequence overlap with MS-associated proteins (Fig. 4A); however, this effect was only found in the 15mer group, whose threshold for peptide sequence overlap is around 50%^17^; this indicates a greater percentage of exact sequence matching and overall homology of SARS-CoV-2 nucleocapsid to MS proteins. In addition, we found that no myelin proteins shared homology with any coronaviruses except for PLP and SARS-CoV-2. Discussion has arisen querying whether the SARS-CoV-2 pandemic will herald an increase in MS incidence^64^. Our results provide a computational basis for this hypothesis, which should be further investigated using epidemiological approaches.

Here, we utilized a new homology-based package called PEPMatch to determine the sequence overlap between immunodominant proteins from SARS-CoV-2 and proteins associated with MS. We found that nucleocapsid significantly overlapped with MS-associated proteins, including PLP. Our work suggests that a variety of proteins may be involved in triggering autoimmunity associated with MS pathogenesis in certain individuals. We chose to focus our analysis on understanding T-cell-driven molecular mimicry, though the creation and maintenance of autoantibodies has been strongly implicated in both MS and COVID-19 severity^38,65^. Recent reports have shown that nucleocapsid is critical in driving humoral immunity in both SARS-CoV^66^ and SARS-CoV-2^67^. Future integrated experimental and computational efforts should focus on understanding the full breadth of autoimmunity following SARS-CoV-2 infection, including the involvement of other organ systems and both adaptive immune arms, for a more comprehensive understanding of pathological sequelae of SARS-CoV-2.

## Methods

### Protein compilation

MS-associated antigens were compiled using the Immune Epitope Database and Analysis Resource (http://www.iedb.org/). The search included the following parameters: “Organism: Homo Sapiens”, “Include Positive Assays”, “No B Cell Assays”, “Disease Data: Multiple Sclerosis (DOID:2377)”, and “MHC Restriction Type: Class I” (class I restriction was added to ensure that many proteins associated with both class I and class II were included in the analysis, as the vast majority of the 1200 + proteins discovered without filtering were associated only with class II). Finally, proteins were added which have been associated strongly with MS in the literature that did not originally appear in the IEDB filtering^68^. This list was trimmed down to proteins which had updated UniProt IDs, leading to the list of 108 MS-associated antigens used in this analysis. This list, with UniProt IDs, can be found in Table 1. Complete list of query proteins in this analysis, including proteins from SARS-CoV-2 and seasonal coronaviruses, as well as EBV and CMV proteins, can also be found in Table 1.

### Homology assessment

Homology between SARS-CoV-2 immunodominant proteins and the list of MS-associated proteins was conducted primarily using PEPMatch and BLASTp. All code utilized in this analysis can be found on GitHub using the following link: https://github.com/mad-scientist-in-training/PEPMatch_SARS-CoV-2_MS.

### PEPMatch

PEPMatch is a homology-based algorithm developed by Daniel Marrama^17^ and is freely available to use on GitHub: https://github.com/IEDB/PEPMatch. PEPMatch was utilized to preprocess the list of MS-associated proteins and query proteins (found in Table 1). Custom python scripts were created to utilize PEPMatch and perform all other processing necessary to run the package (see Homology Assessment for link to GitHub page). Parameters were set to preprocess all data sets separately into 9mer peptides and 15mer peptides, with 2 and 7 mismatches respectively. PEPMatch output for all significant tests is available in Supplemental Table 1.

### BLASTp

To determine homology between PEPMatch-predicted peptides overlapping with nucleocapsid and experimentally validated epitopes (Fig. 2A), a list of epitopes for each protein was compiled using the IEDB. The search parameters included “Antigen: Protein_of_interest (Homo sapiens (human))”, “Include Positive Assays”, and “Host: Homo sapiens (human)”. Standard Protein BLAST (BLASTp) was used to determine the homology between all PEPMatch-predicted epitopes and experimentally validated peptides for each protein. To normalize, the total number of homology “hits” returned from BLASTp was divided by the number of peptides predicted from PEPMatch for each protein.

Example BLASTp analysis: **PLP.**

**Table:** 

Peptides returned from PEPMatch (overlapping with nucleocapsid):	1
Epitopes returned from IEDB query:	150
Number of Homology “hits” from BLASTp analysis	20
Normalized total hits	20/1 = 20

BLASTp was also used to determine the percent identity between nucleocapsid from SARS-CoV-2 and seasonal coronaviruses using the standard, recommended parameters.

### Statistics

For statistical comparisons, each query protein (spike, nucleocapsid, and membrane, NS7a, replicase polyprotein1ab, and envelope) was separately processed to provide “background” number of hits against MS-associated proteins. Specifically, custom python scripts (found on the GitHub page) were created to shuffle the amino acid sequence of each query protein, which were run to determine the number of matches with MS-associated proteins as background matching signal. Duplicate matches (for example, if identical peptides from 2 separate MS-associated proteins matched a SARS-CoV-2-derived peptide) were removed prior to statistical testing. For each query protein tested, 30 iterations of unique shuffled comparisons were run, which were then averaged and used in a Fisher’s exact or Chi-square test for statistical comparison. All analyses were run conservatively with two-tailed parameters; however, if the number of random matches exceeded the number of matches of the intact peptide, the *p* value was assumed to be 1. The following is an example of the statistics utilized for each protein in this analysis:

**Spike, SARS-CoV-2**

**Table:** 

9mer peptides	Match in MS proteins	No match in MS proteins	Total
Intact Peptides	12	1253	1265
Shuffle Peptides (avg)	5	1260	1265

*p* = 0.1421 (− log_10_ transform: 0.847).

**Table:** 

15mer peptides	Match in MS Proteins	No Match in MS Proteins	Total
Intact Peptides	66	1193	1259
Shuffle Peptides (avg)	48	1211	1259

*p* = 0.1028 (− log_10_ transform: 0.988).

Statistical analyses (as above) for all comparisons in this manuscript can be found in Supplemental Table 2.

### 3D structure of PLP

Structural visualization of myelin proteolipid protein (PLP) was conducted using AlphaFold^69^ and ChimeraX^70,71^. Specifically, AlphaFold-simulated structure of PLP (ID: P60201) was loaded into ChimeraX for manipulation and visualization. Protein regions of PLP identified to be immunodominant, specifically regions 40–60, 95–117, 117–150, and 185–206^20^, were highlighted and colored for accentuation. The sequence of PLP used throughout the analysis can be found on UniProt (https://www.uniprot.org/).

### Allele assessment

The MHC binding prediction machine learning algorithm from the IEDB was used to determine whether MS-associated alleles were predicted to preferentially bind and present nucleocapsid peptides in comparison to a reference set of alleles. MS-associated alleles were curated from the literature^22–24^ and used in comparison to an allele set from the IEDB that covers 99% of the population^25^. Nucleocapsid peptide “hits” from PEPMatch were used in the query against the total set of alleles (MS and reference) using the IEDB recommended 2.22 standard parameter. As most binding predictions were skewed heavily towards high percentile ranks, the top 50% percentile of all matching predictions were focused on to capture information for all alleles input while also providing physiologically relevant binding information. In Fig. 3C, the nucleocapsid peptide-MHC binding predictions of percentile rank 10 or less^26^ were cross-referenced with the original PEPMatch output to determine the homologous MS-associated proteins, and the frequency of peptide matches were tabulated and graphed.

### Graphics

Graphics in this article were created using Biorender (https://biorender.com/).

## Supplementary Information

Supplementary Information 1.

Supplementary Table 1.

## Data Availability

The authors declare that all data in support of the main findings of this study are available within the paper and its supplementary information files. All other data (including raw data generated in the supplemental findings) are available upon reasonable request to the corresponding author.

## Code availability

All code generated for this project is available on GitHub at the following repository: https://github.com/mad-scientist-in-training/PEPMatch_SARS-CoV-2_MS.
