# Supplementary material for: Sequence similarity between SARS-CoV-2 nucleocapsid and multiple sclerosis-associated proteins provides insight into viral neuropathogenesis following infection
Source: Sci Rep. 2023 Jan 8;13:389. doi: 10.1038/s41598-022-27348-8 (PMC9825799; doi:10.1038/s41598-022-27348-8)
Supplement: Supplementary file 1 — Supplementary Information 1. [file 41598_2022_27348_MOESM1_ESM.pdf]

Supplemental Information Title & Legends

Supplemental Figure 1: EBV immunodominant antigens show increased homology with MS-associated proteins in comparison to CMV antigens. The same analysis was performed as in Figure 1A, using EBV[69] and CMV[70] immunodominant epitopes.

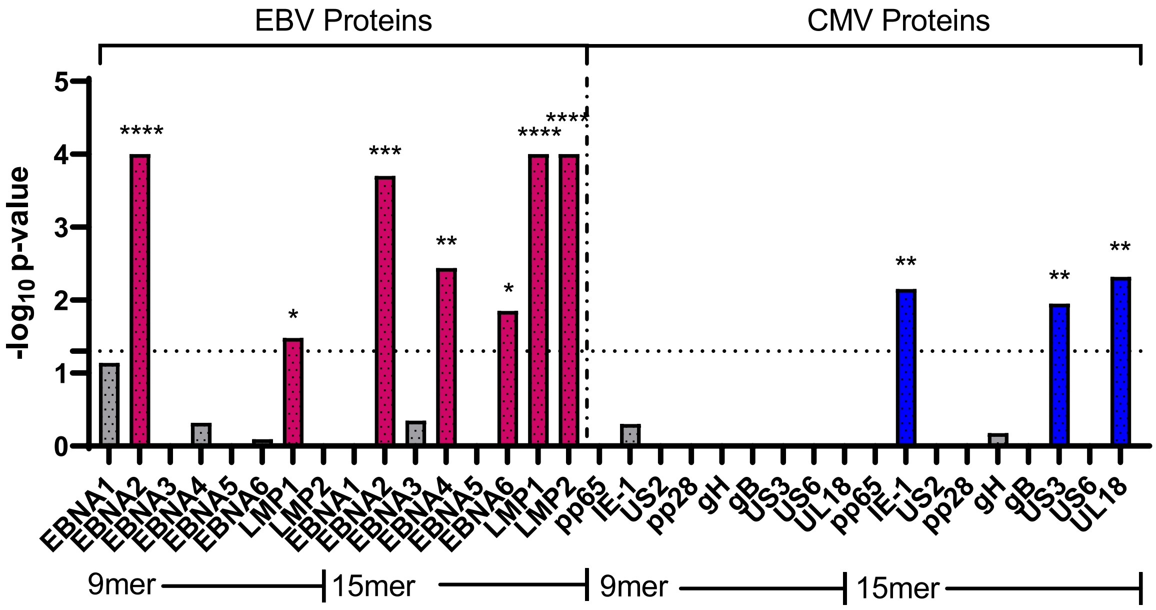

Supplemental Table 1: PEPMatch output from significant tests.

This table contains all of output from significant PEPMatch runs found in the main manuscript, including results from SARS-CoV-2 and seasonal coronaviruses. Data includes pathogen, matching peptide sequences and lengths, gene names, protein IDs, the number of mismatches and mutated positions. Note that duplicate peptide sequences for a single test were deleted prior to statistical testing, which is not reflected in this table.

Supplemental Table 2: Raw values of PEPMatch output for statistical testing.

This table contains all values post processing utilized in Fisher's exact testing for statistical analysis.

Supplemental Table 2: Raw Values of PEPMatch Output for Statistical Testing

Organism	Protein	Protein Abbreviation	Peptide Length	Total Matches	Ave Random Matches	Total Possible Matches	p Value	Log_{10} Transform
SARS-CoV-2	Spike	S	9mer	12	5	1265	0.1421	0.847405922
SARS-CoV-2	Spike	S	15mer	66	48	1259	0.1028	0.988006885
SARS-CoV-2	Membrane	M	9mer	0	1	214	1	0
SARS-CoV-2	Membrane	M	15mer	6	10	208	1	0
SARS-CoV-2	Nucleocapsid	N	9mer	13	3	411	0.0201	1.696803943
SARS-CoV-2	Nucleocapsid	N	15mer	48	21	405	0.0009	3.045757491
SARS-CoV-2	NS7a	7a	9mer	1	1	113	1	0
SARS-CoV-2	NS7a	7a	15mer	1	5	107	1	0
SARS-CoV-2	R1AB/ORF1ab	rep	9mer	37	32	7088	0.6293	0.201142268
SARS-CoV-2	R1AB/ORF1ab	rep	15mer	297	251	7082	0.0499	1.301899454
SARS-CoV-2	Envelope	E	9mer	2	1	67	1	0
SARS-CoV-2	Envelope	E	15mer	2	4	61	1	0
HCoV-229E	Spike	S	9mer	6	6	1165	1	0
HCoV-229E	Spike	S	15mer	46	47	1159	1	0
HCoV-229E	Membrane	M	9mer	5	1	217	0.2155	0.666552726
HCoV-229E	Membrane	M	15mer	16	6	211	0.0465	1.332547047
HCoV-229E	Nucleocapsid	N	9mer	5	2	381	0.451	0.345823458
HCoV-229E	Nucleocapsid	N	15mer	32	21	375	0.1536	0.813608784
HCoV-NL63	Spike	S	9mer	4	6	1348	1	0
HCoV-NL63	Spike	S	15mer	40	50	1342	1	0
HCoV-NL63	Membrane	M	9mer	0	1	218	1	0
HCoV-NL63	Membrane	M	15mer	9	9	212	1	0
HCoV-NL63	Nucleocapsid	N	9mer	4	2	369	0.6862	0.163549286
HCoV-NL63	Nucleocapsid	N	15mer	46	19	363	0.0006	3.22184875
HCoV-OC43	Spike	S	9mer	9	7	1345	0.803	0.095284455
HCoV-OC43	Spike	S	15mer	55	42	1339	0.2144	0.668775219
HCoV-OC43	Membrane	M	9mer	0	1	222	1	0
HCoV-OC43	Membrane	M	15mer	5	5	216	1	0
HCoV-OC43	Nucleocapsid	N	9mer	2	2	440	1	0
HCoV-OC43	Nucleocapsid	N	15mer	51	20	434	0.0002	3.698970004
HCoV-HKU1	Spike	S	9mer	5	6	1348	1	0
HCoV-HKU1	Spike	S	15mer	51	48	1342	0.8378	0.076859644
HCoV-HKU1	Membrane	M	9mer	0	1	215	1	0
HCoV-HKU1	Membrane	M	15mer	11	7	209	0.4708	0.327163546
HCoV-HKU1	Nucleocapsid	N	9mer	6	2	433	0.2868	0.542420853
HCoV-HKU1	Nucleocapsid	N	15mer	66	17	427	0.0001	4
Epstein-Barr virus (strain B95-8)	Epstein-Barr nuclear antigen 1	EBNA1	9mer	77	57	418	0.073	1.13667714
Epstein-Barr virus (strain B95-8)	Epstein-Barr nuclear antigen 1	EBNA1	15mer	234	307	486	1	0
Epstein-Barr virus (strain B95-8)	Epstein-Barr nuclear antigen 2	EBNA2	9mer	32	8	457	0.0001	4
Epstein-Barr virus (strain B95-8)	Epstein-Barr nuclear antigen 2	EBNA2	15mer	119	72	462	0.0002	3.698970004
Epstein-Barr virus (strain B95-8)	Epstein-Barr nuclear antigen 3	EBNA3	9mer	7	7	930	1	0
Epstein-Barr virus (strain B95-8)	Epstein-Barr nuclear antigen 3	EBNA3	15mer	64	55	930	0.4483	0.348431261
Epstein-Barr virus (strain B95-8)	Epstein-Barr nuclear antigen 4	EBNA4	9mer	11	7	917	0.4785	0.320118058
Epstein-Barr virus (strain B95-8)	Epstein-Barr nuclear antigen 4	EBNA4	15mer	87	53	917	0.0036	2.443697499
Epstein-Barr virus (strain B95-8)	Epstein-Barr nuclear antigen leader protein	EBNA5	9mer	3	10	111	1	0
Epstein-Barr virus (strain B95-8)	Epstein-Barr nuclear antigen leader protein	EBNA5	15mer	15	82	111	1	0
Epstein-Barr virus (strain B95-8)	Epstein-Barr nuclear antigen 6	EBNA6	9mer	9	7	954	0.8028	0.095392636
Epstein-Barr virus (strain B95-8)	Epstein-Barr nuclear antigen 6	EBNA6	15mer	85	56	965	0.0141	1.850780887
Epstein-Barr virus (strain B95-8)	Latent membrane protein 1	LMP1	9mer	12	3	351	0.0333	1.477555766
Epstein-Barr virus (strain B95-8)	Latent membrane protein 1	LMP1	15mer	67	25	357	0.0001	4
Epstein-Barr virus (strain B95-8)	Latent membrane protein 2	LMP2	9mer	4	5	489	1	0
Epstein-Barr virus (strain B95-8)	Latent membrane protein 2	LMP2	15mer	79	39	483	0.0001	4
Human cytomegalovirus (strain AD169)	65 kDa phosphoprotein	pp65	9mer	4	4	553	1	0
Human cytomegalovirus (strain AD169)	65 kDa phosphoprotein	pp65	15mer	11	20	547	1	0
Human cytomegalovirus (strain AD169)	Immediate early protein IE1	IE-1	9mer	6	3	483	0.5058	0.296021175
Human cytomegalovirus (strain AD169)	Immediate early protein IE1	IE-1	15mer	48	25	477	0.007	2.15490196
Human cytomegalovirus (strain AD169)	Envelope glycoprotein B	gB	9mer	5	5	898	1	0
Human cytomegalovirus (strain AD169)	Envelope glycoprotein B	gB	15mer	34	33	892	1	0
Human cytomegalovirus (strain AD169)	Unique short US2 glycoprotein	US2	9mer	0	0	191	1	0
Human cytomegalovirus (strain AD169)	Unique short US2 glycoprotein	US2	15mer	3	6	185	1	0
Human cytomegalovirus (strain AD169)	Cytoplasmic envelopment protein 3	UL99	9mer	2	1	182	1	0
Human cytomegalovirus (strain AD169)	Cytoplasmic envelopment protein 3	UL99	15mer	13	10	176	0.6671	0.175809059
Human cytomegalovirus (strain AD169)	Envelope glycoprotein H	gH	9mer	4	5	735	1	0
Human cytomegalovirus (strain AD169)	Envelope glycoprotein H	gH	15mer	34	35	729	1	0
Human cytomegalovirus (strain AD169)	Membrane glycoprotein US3	US3	9mer	0	0	178	1	0
Human cytomegalovirus (strain AD169)	Membrane glycoprotein US3	US3	15mer	19	6	172	0.0113	1.946921557
Human cytomegalovirus (strain AD169)	Unique short US6 glycoprotein	US6	9mer	0	1	175	1	0
Human cytomegalovirus (strain AD169)	Unique short US6 glycoprotein	US6	15mer	3	7	169	1	0
Human cytomegalovirus (strain AD169)	Glycoprotein UL18	UL18	9mer	3	2	360	1	0
Human cytomegalovirus (strain AD169)	Glycoprotein UL18	UL18	15mer	26	9	354	0.0048	2.318758763
